# Exploring the Use of Amine Modified Mesoporous Magnesium Carbonate for the Delivery of Salicylic Acid in Topical Formulations: In Vitro Cytotoxicity and Drug Release Studies

**DOI:** 10.3390/molecules24091820

**Published:** 2019-05-11

**Authors:** Maria Vall, Natalia Ferraz, Ocean Cheung, Maria Strømme, Teresa Zardán Gómez de la Torre

**Affiliations:** Division for Nanotechnology and Functional Materials, Department of Engineering Sciences, SE-751 21 Uppsala, Sweden; maria.vall@angstrom.uu.se (M.V.); natalia.ferraz@angstrom.uu.se (N.F.); ocean.cheung@angstrom.uu.se (O.C.); maria.stromme@angstrom.uu.se (M.S.)

**Keywords:** mesoporous, magnesium carbonate, amine functionalization, cytotoxicity, salicylic acid, drug release

## Abstract

Salicylic acid (SA) has for a long time been used to treat various skin disorders due to its anti-inflammatory, bacteriostatic, and antifungal properties. In the present work, mesoporous magnesium carbonate (MMC), a promising drug carrier, was modified with 3-aminopropyl-triethoxysilane to enable loading of SA. The amine modified MMC (aMMC) was successfully loaded with 8 wt.% of SA via a solvent evaporation method. SA was later completely released from the carrier in less than 15 min. Furthermore, the cytotoxicity of the functionalized material was evaluated. aMMC was found to be non-toxic for human dermal fibroblast cells with particle concentration of up to 1000 µg/mL when exposed for 48 h. The presented results form the basis of future development of aMMC as a potential carrier for SA in dermatological applications.

## 1. Introduction

Salicylic acid (SA) has been used as a peeling agent in order to treat skin disorders for more than 2000 years [1]. SA can be synthesized or found naturally in the bark of willow and in the leaves of wintergreen. SA has a comedolytic effect which can resolve or prevent the formation of comedones (plugged hair follicles) [2]. It also possesses a keratolytic effect [2,3], which is the ability to break down and dissolve the outermost layer of skin (stratum corneum, that consists of dead skin cells). SA blocks the in vivo production of pro-inflammatory prostaglandins, giving it anti-inflammatory properties, and it has bacteriostatic as well as fungicidal properties [3,4,5]. These properties make SA useful as a peeling agent for patients suffering from acne, a common skin disease that occurs when hair follicles become plugged with dead skin cells and sebum [6]. It is also used when treating post-acne erythema and hyperpigmentation of skin, which is a consequence of acne. SA also has the ability to decrease the secretion of sebum, which adds to its therapeutic effect [7]. Being a lipophilic agent, SA easily penetrates the skin where the therapeutic effect is desired [2]. Apart from acne, SA has also been proven to be effective toward other types of skin conditions like psoriasis [8,9]. Different carrier materials have been used for both topological and systemic administration of SA, for example, it has been loaded into the polymer backbone in biodegradable polymers (e.g., cross-linked polyester based on xylitol and adipic acid) for a sustained release of SA [10]. SA has also been loaded into different porous matrices, in MCM-41 and SBA-15 [11] and also in halloysite nanotubes functionalized with dendrimers [12].

In 2013, our group published the synthesis of a mesoporous magnesium carbonate material (MMC) with a high surface area (>700 m^2^/g, dependent on synthesis parameters) and a narrow pore size distribution centered at ~5 nm [13,14,15]. Magnesium carbonate is listed as GRAS (generally recognized as safe) by the U.S. Food and Drug Administration (FDA) and is used as an additive in food (E504) [16,17]. Moreover, we have previously demonstrated that the mesoporous form of the material (MMC) is non-toxic toward human dermal fibroblast cells; it promoted a negligible cutaneous reaction in a rabbit skin irritation model and displays antibacterial properties [18,19]. MMC has been proven to be a very promising drug delivery vehicle; it has the capability of stabilizing poorly soluble active pharmaceutical ingredients (APIs) in their amorphous form within the pore structure and, hence, significantly enhances the apparent solubility of the APIs [15,20,21,22].

All the above-mentioned properties make MMC an attractive material in dermatological applications. The direct application of MMC as a carrier of SA is problematic due to the basic and acidic natures of the MMC and SA, respectively. SA contains acidic hydroxyl groups (pKa 2.97) that would readily react with the carbonate groups on MMC. One way to avoid such a chemical reaction is to functionalize the surface of MMC with aminosilanes. Surface functionalization using aminosilanes is one of the most commonly used methods for post-synthesis functionalization of mesoporous materials. Grafting aminosilanes to the surface of different porous materials has been executed to improve their performance in CO_2_ sorption [23,24], drug delivery [25,26], and catalysis [27]. Surface modification also allows the drug loading and release profile of porous materials to be tuned [28]. We have earlier reported on the synthesis and post-functionalization of MMC with (3-aminopropyl)triethoxylsilane (APTES) [29]. It was demonstrated that the amine grafting on the surface of MMC could be used to control the release rate of loaded ibuprofen [29]. Amine-modified MMC (aMMC) also showed enhanced azo dye uptake and was more stable in a moist environment when compared to the non-functionalized material [30,31].

In this work, we investigate the possibility of using aMMC as a carrier of SA in dermatological applications. The in vitro cytotoxicity of the material is analyzed using human dermal fibroblast (hDF) cells and the loading and release of SA from aMMC is investigated.

## 2. Results and Discussion

### 2.1. Material Characterization

The specific surface area (S_BET_), pore diameter, pore volume, and the particle size of the studied samples are given in Table 1. Noticeably, the particle size was reduced after the amine grafting. This is believed to be due to disintegration of MMC agglomerates into smaller aMMC particles during the functionalization process. From the table, it is clear that the two unloaded samples (MMC and aMMC) were highly porous where the amine functionalized material had slightly lower pore volume and pore diameter as a consequence of the amine grafting. After loading aMMC with SA (aMMC–SA), the surface area and pore volume were reduced even further. aMMC–SA still remained highly porous, which is an indication that SA was loaded inside the porous structure rather than on the surface. If SA was loaded on the surface of the material, the pores of aMMC would have been blocked by the loaded SA and, therefore, the pore volume of aMMC would have been lowered noticeably. To further attest to the fact that SA was loaded inside the pores, we estimated the theoretical pore volume when 8 wt.% SA was loaded onto the inside of the pores on aMMC. This estimated value was compared with the experimental value of aMMC loaded with 8 wt.% SA as well as the theoretical value for the pore volume of aMMC mixed with 8 wt.% SA. The theoretical value of loading inside the pores was close to the measured value (supporting information Table S1), which also is an indication that SA was primarily loaded inside the pores of aMMC.

**Figure 1:**
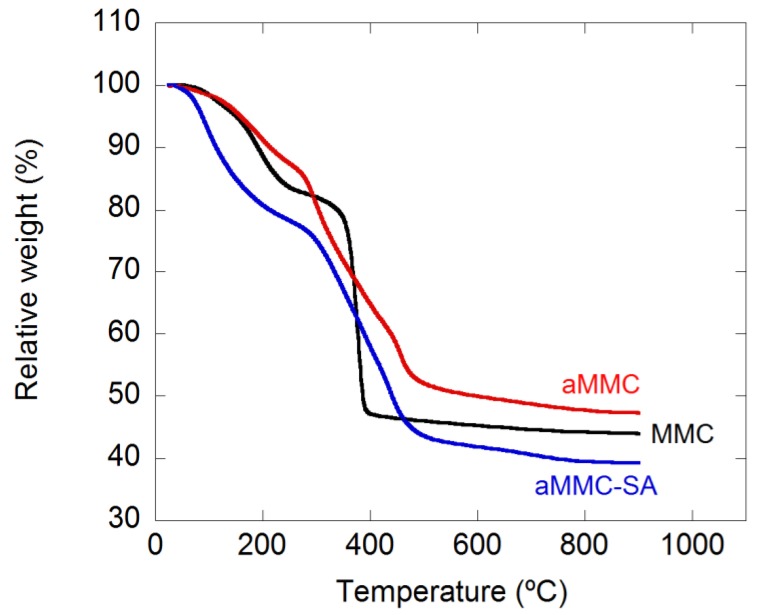
TGA curves for the studied samples.

Thermal gravimetric analysis (TGA) curves for MMC, aMMC, and aMMC–SA are presented in Figure 1. A significant drop in mass at around 400 °C was observed for MMC, which can be attributed to the loss of CO_2_. For aMMC and aMMC–SA, a more prolonged drop in mass between 300 °C and 500 °C can be seen. This mass drop is also attributed to the decomposition of the carbonate. The prolonged decomposition is due to the addition of the amine groups and is in good agreement with previous results [29]. The weight loss of aMMC–SA was compared with the weight loss of aMMC and it was found that the amount of loaded SA in aMMC was about 8 wt.%.

**Figure 2:**
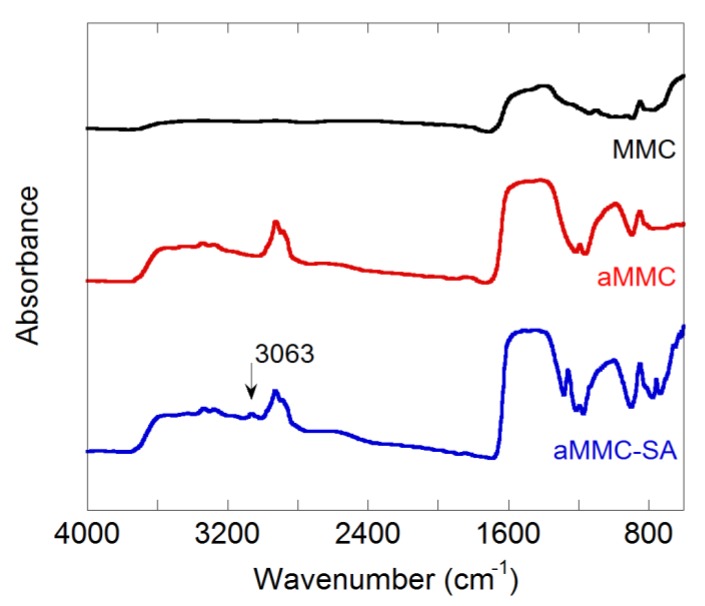
FTIR absorption spectra for the samples under study. Note that the carbonate band at around 1400 cm^−1^ is saturated on aMMC and aMMC–SA and, therefore, its intensity should not be considered quantitatively.

Fourier transform infrared spectroscopy (FTIR) spectra were recorded for MMC, aMMC, and aMMC–SA (see Figure 2). IR spectra of MMC and aMMC have been discussed in detail previously [13,29]. The three materials exhibited a broad band at 1440 cm^−1^ and a sharp band at 850 cm^−1^ which were attributed to vibrations of the carbonate group. For aMMC and aMMC–SA there were two bands visible at 3300 and 3250 cm^−1^, which can be attributed to the stretch of the N–H stretching vibration mode of the grafted APTES molecules. There were two bands just below 3000 cm^−1^, which are attributed to the C–H stretching vibration mode of the APTES molecule. For aMMC–SA, there was also a band at 3063 cm^−1^, which is related to the stretching vibration of aromatic C–H bonds present in the structure of SA. This band also confirms that SA was loaded into the sample.

**Figure 3:**
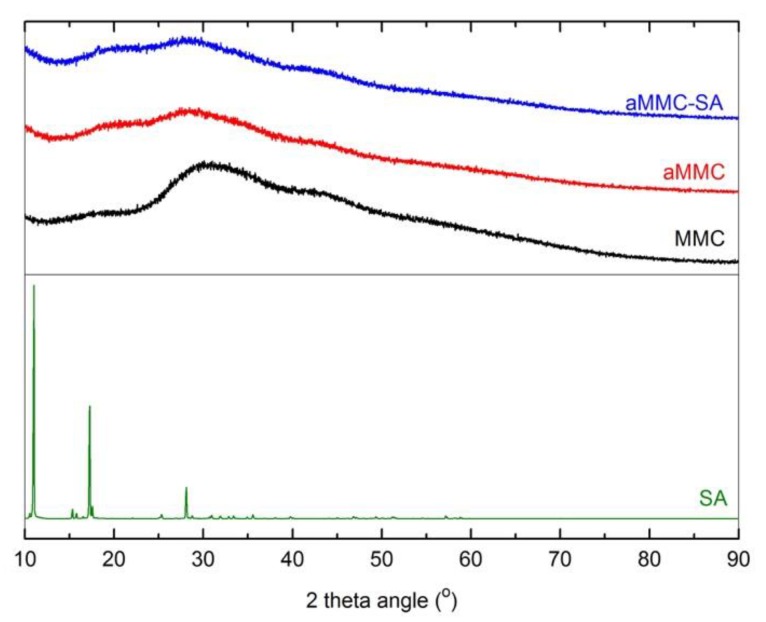
XRD patterns for MMC, aMMC, aMMC–SA, and pure SA.

**Figure 4:**
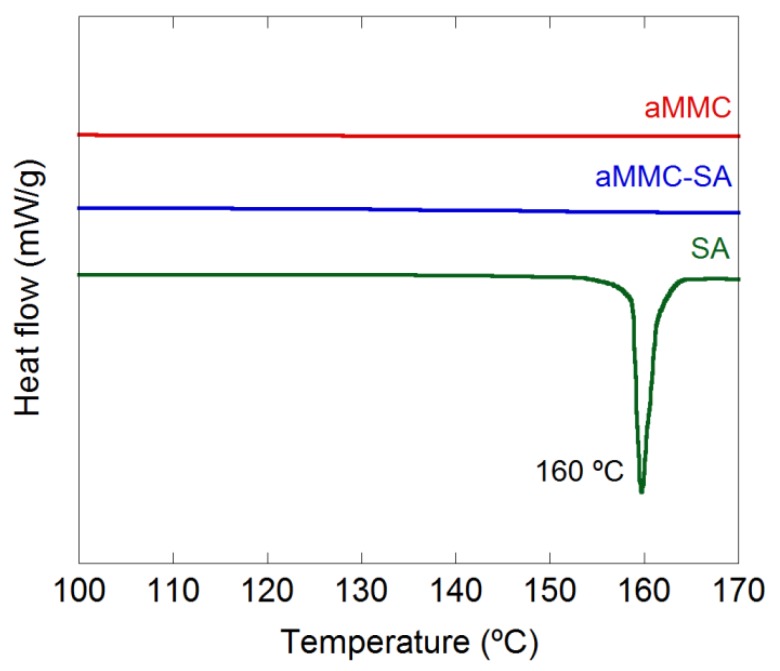
DSC thermograms for aMMC, aMMC–SA, and pure SA.

**Figure 5:**
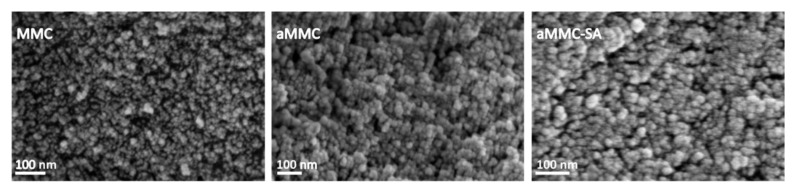
SEM images of MMC, aMMC, and aMMC-SA.

Figure 3 and Figure 4 show the X-ray powder diffraction (XRD) patterns and the differential scanning calorimetry (DSC) curves, respectively, for all studied samples. From the figure displaying the XRD patterns, it is clear that all samples were X-ray amorphous. The absence of peaks corresponding to crystalline SA in the XRD spectrum indicates that the loaded SA was in a non-crystalline state. This supports the results from N_2_ adsorption measurements, showing a reduction in pore volume after loading, indicating that the SA had actually entered the aMMC pore structure. The non-crystalline state of the SA was also confirmed by DSC where the endothermic peak at 160 °C, corresponding to the melting point of crystalline SA, was absent in the loaded aMMC sample (Figure 4). It is worth mentioning that there were no observable changes on the morphology of aMMC after SA loading (Figure 5).

### 2.2. In Vitro Cytotoxicity

The cytotoxic effect of aMMC on hDF was investigated using the alamar blue assay. The cells were exposed to different concentrations of the material (1000, 500, 200, and 50 μg/mL) during either 24 or 48 ± 2 h. Previously, it has been demonstrated that unmodified MMC with a size distribution of 50–200 μm and a specific surface area of 207 m^2^/g had no toxic effect on the same type of cells [18].

**Figure 6:**
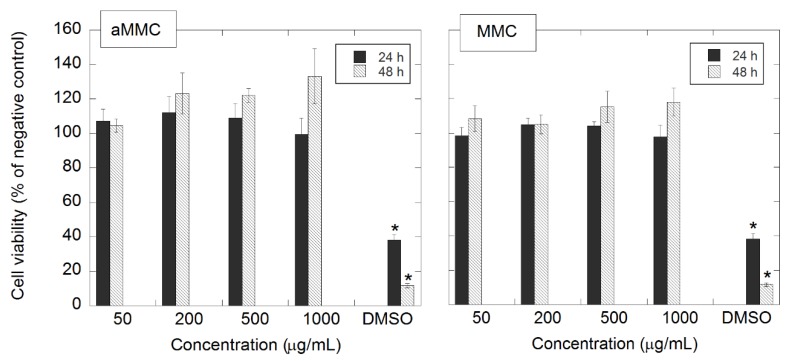
Cell viability of hDF cells exposed to aMMC (left panel) and MMC (right panel) after 24 and 48 h. The error bars represent the standard error of the mean and are based on *n* = 6. Statistically significant differences as compared to the negative control are marked with * (*p* < 0.05).

**Figure 7:**
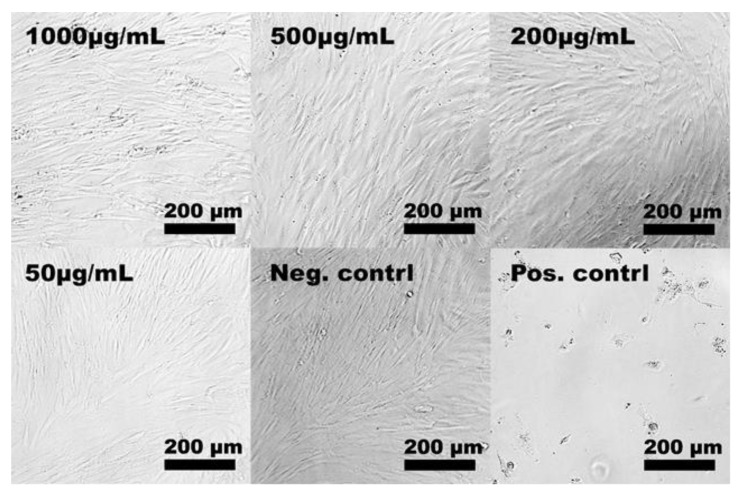
Representative light microscope images of hDF cells exposed to different particle concentrations of aMMC for 48 h. The negative and the positive control samples represent untreated cells and cells that have been exposed to 5% DMSO, respectively.

**Figure 8:**
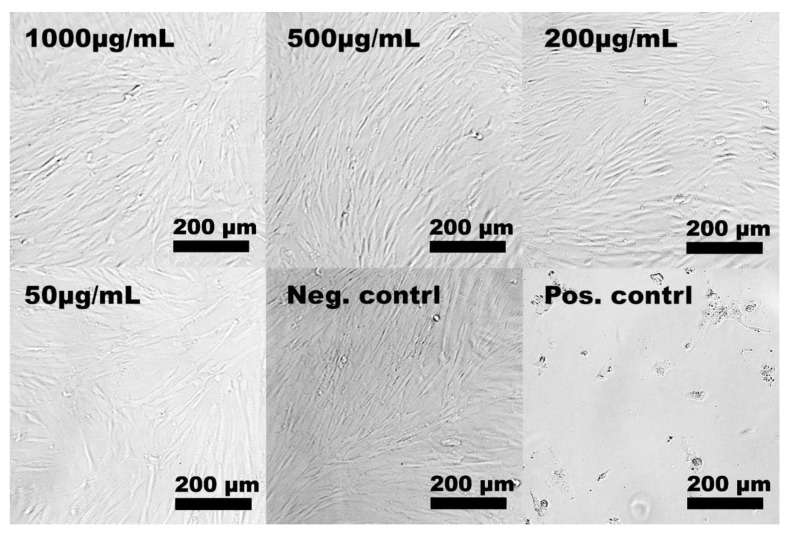
Representative light microscope images of hDF cells exposed to different particle concentrations of MMC for 48 h. The negative and the positive control samples represent untreated cells and cells that have been exposed to cell culture medium and 5% DMSO, respectively.

In this study, the starting material for the synthesis of aMMC was MMC with different characteristics in terms of particle size distribution and specific surface area compared with the MCC material studied by Frykstrand et al. [18]. Since such properties together with surface chemistry are known to influence cell–material interactions and, in turn, the potential toxicity of the material [32], MCC particles (as-synthesized, before chemical modification) were included in the cytotoxicity studies of the amine-grafted particles. The cell viability after exposure to both materials is presented in Figure 6. After 24 and 48 h of exposure to aMMC (left panel) and MMC (right panel), no significant differences in cell viability between treated and non-treated cells (negative control) were found. Thus, none of the MMC materials exhibited cytotoxic effects up to 1000 μg/mL. The light microscopy results confirm the non-toxic effect of aMMC and MMC. From Figure 7 and Figure 8 it can be observed that the cells adhere in a larger number to the tissue culture plate and exhibit a cell morphology comparable to the negative control up to 48 h.

Thus, the non-toxic profile of MCC is confirmed here, where particles with higher surface area and broader particle size distribution than previously investigated MCC materials were assessed. Moreover, the post-functionalization of MCC with APTES did not alter the safety profile of the MCC material toward the dermal cells.

### 2.3. Drug-Release Test

**Figure 9:**
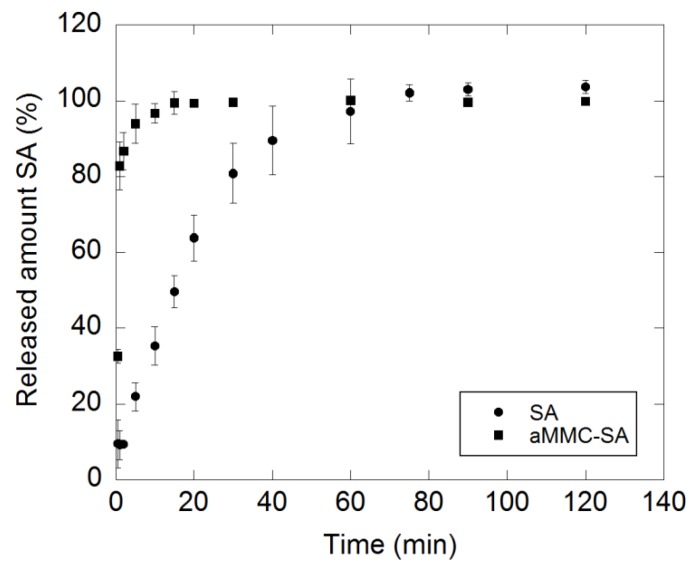
Dissolution profile of crystalline SA as well as release and concomitant dissolution profile of SA incorporated in aMMC. All measurements were made in triplicates and the data is displayed as the mean values with corresponding standard deviation.

The dissolution profile of crystalline SA and the release and concomitant dissolution of SA loaded in aMMC are displayed in Figure 9. The release and dissolution of the loaded SA was more rapid than the dissolution of the crystalline substance. During the first minute, the release and dissolution of the loaded SA was almost nine times higher than the dissolution of the pure SA. The loaded SA was completely released from the carrier and dissolved within 15 min, while it took 75 min for the pure substance to completely dissolve. Fast dissolution of the loaded substance was due to the non-crystalline state of the loaded SA. The observed fast release and dissolution of the loaded substance showed a diffusion process of the SA within the pore structure of the carrier that did not severely hinder the release [33]. The profiles eventually levelled out at the same concentration (80 mg/L).

The results presented here show that SA incorporated into aMCC results in faster release and dissolution when tested in vitro, as compared to the dissolution of pure crystalline SA. A rapid release of SA may be beneficial for antimicrobial and anti-inflammatory applications such as treatment of infections, which is desired in patients suffering from acne. The non-cytotoxic effect on hDF and the amount of SA that was successfully loaded in aMMC (8 wt.%) shows the potential of using aMMC as a carrier of SA that can possibly be used in dermatological formulations. Over-the-counter preparations normally contain 0.5%–5% SA, whereas prescription formulations for treatment against more severe conditions can contain SA concentrations of 6% or more [3]. The amount of SA in a formulation with aMMC will depend on the overall composition.

## 3. Materials and Methods

### 3.1. Synthesis of MMC

The synthesis of MMC has previously been described in detail [13,15] but in short; 20 g of MgO and 300 mL of MeOH were mixed and sealed in a reaction vessel and pressurized with 4 bar of CO_2_ and left for 24 h under constant stirring at room temperature. The reaction mixture was then centrifuged at 3374× *g* for 30 min in order to separate the unreacted MgO from the synthesis liquid. The resulting gel was dried at 0 °C under constant stirring until white powder was formed.

### 3.2. Amine Modification of MMC

Aminopropyl-(3-ethoxysilane) was grafted to the surface of MMC following the protocol previously described by us [29]. The reaction was carried out under dry conditions. All glassware was dried over night at 150 °C and the reaction was performed under N_2_ atmosphere. 5 g of MMC were dispersed in 300 mL of toluene and afterward heated to 110 °C. 8.5 mmol/g of APTES were added to the solution and the reaction was then carried out for 24 h under reflux. The modified MMC was filtered off and washed twice with 50 mL EtOH and finally dried overnight at 70 °C.

### 3.3. Material Characterization

#### 3.3.1. N_2_ Sorption Analysis

The Brunauer–Emmett–Teller (BET) specific surface area (S_BET_) and the porosity of the materials were determined by recording nitrogen adsorption and desorption isotherms (at 78 K) using a Micromeritics ASAP 2020 surface area analyzer (Norcross, GA, USA). Prior to the analysis, the samples were pre-treated by heating to 373 K under a dynamic vacuum (1 × 10^−4^ Pa) using a Micromeritics SmartVacPrep 067 sample preparation unit. Equilibrium adsorption data points were obtained when the change in pressure dropped below 0.01% within a 10 s interval (with minimum 100 s delay). The S_BET_ was obtained using the BET equation [34] for adsorption points between p/p_0_ = 0.05 and 0.15.

#### 3.3.2. X-ray Powder Diffraction (XRD)

XRD patterns were recorded using a Bruker D8 Twin Twin diffractometer (Bruker, Billerica, MA, USA) with Cu–K*_α_* radiation (λ = 1.54 Å) for 2θ = 10.0 to 90.0° at room temperature. The instrument was set to operate at 45 kV and 40 mA.

#### 3.3.3. Differential Scanning Calorimetry (DSC)

DSC was performed on a DSC Q2000 instrument (TA Instrument, New Castle, DE, USA). Samples of 3.2–5.5 mg were weighed into 5 mm aluminum pans and sealed hermetically. The samples were first cooled down to −35 °C and then heated to 180 °C at a heating rate of 10 °C/min. The instrument was calibrated for the melting point and heat of fusion of indium (156.6 °C and 28.4 mJ/mg).

#### 3.3.4. Thermal Gravimetric Analysis (TGA)

Samples were heated from 25 to 900 °C with a heating rate of 10 °C/min under a constant flow of air (20 mL/min), using a Mettler Toledo TGA2 (Mettler Toledo, Schwerzenbach, Switzerland).

#### 3.3.5. Fourier Transform Infrared Spectroscopy (FTIR)

IR spectra were obtained using a Varian 670-IR Fourier transform IR spectrometer (Varian, Santa Clara, CA, USA) coupled with a Varian 670-IR IR microscope and a Linkam THM-600 (Tadworth, UK) heating stage (room temperature to 600 °C). The samples were heat-treated to 150 °C in situ under dry nitrogen atmosphere to remove adsorbed water before data collection. Transmission IR spectra of the water-free samples were recorded for a sample area of 100 × 100 μm^2^ (600–4000 cm^−1^ with 4 cm^−1^ resolution). A mercury–cadmium–telluride (MCT) detector was used to record the IR spectra.

#### 3.3.6. Scanning Electron Microscopy (SEM)

The morphology of the samples was examined using Zeiss LEO 1550 and 1530 electron microscopes (Oberkochen, Germany; operated at 2kV) and an in-lens secondary electron detector was used for imaging. Samples were mounted on aluminum stubs with double adhesive carbon tape and sputtered with Au/Pd prior to analysis to avoid charge build-up in the non-conductive materials.

### 3.4. In Vitro Cytotoxicity

Human dermal fibroblasts (hDF, European Collection of Authenticated Cell Cultures ECACC) suspended in DMEM-F12 medium (Thermo Fisher Scientific, Uppsala, Sweden), supplemented with 10% fetal bovine serum, 100 IU/mL penicillin, and 100 μg/mL streptomycin were seeded in 96-well tissue culture plates at a density of 12,500 cells/well. The cells were culture for 24 ± 2 h in a humidified atmosphere at 37 °C and 5% CO_2_, reaching near confluency. The cell culture medium was removed and replaced with new cell culture medium containing 1000, 500, 250, and 50 μg/mL of either MMC or aMMC. The cells were incubated for an additional 24 or 48 ± 2 h at 37 °C and 5% CO_2_ before cell morphology and cell viability were analyzed using light microscopy and the alamar blue assay, respectively. Cell culture medium containing 5% DMSO was used as the positive control and non-treated cells were the negative control. All the samples were run in triplicates.

#### 3.4.1. Alamar Blue Assay

A microplate alamar blue assay was used to measure the cell metabolic activity as an indicator of cytotoxicity after exposure to MMC and aMMC. After either 24 or 48 ± 2 h, the cell culture medium containing the materials was removed from the wells and cells were subsequently washed with PBS. The alamar blue dye was diluted in cell culture medium by a factor of 10 and 200 μL of the mixture was added to the cells. The cells were subsequently incubated for 90 min at 37 °C and 5% CO_2_. Aliquots of 100 μL from each well were transferred to a black 96-well plate and the florescence intensity at 590 nm (excitation wavelength at 560 nm) was recorded using an Infinite^®^ 200 fluorometer (Tecan, Sweden)

A control measurement was performed in order to investigate if there was any interaction between the materials and the alamar blue reagent. The assay was in this case performed with cell culture medium containing either MMC or aMMC in the absence of cells and the fluorescence signal was compared to the negative control. None of the MCC materials interfered with the alamar blue reagent.

#### 3.4.2. Light Microscopy

Light microscopy was used to evaluate the cell morphology of the cells after they were exposed to both materials. After either 24 or 48 ± 2 h, the cell culture medium containing the materials was removed from the wells and the cells were subsequently washed once with PBS and observed under a light microscope (Nikon Eclipse TE2000-U). The image adjustments were made manually in Adobe Photoshop CS6. All images were converted to black and white using a black and white adjustment layer and the contrast was enhanced using adjustment layers for levels and curves.

#### 3.4.3. Statistical Analysis

Data were analyzed by Welch’s ANOVA and Games-Howell post-hoc test using R studio v.3.5.2. Normal distribution of the data was evaluated by Shapiro–Wilk test and equal variances were evaluated by Levenes test. *p*-values lower than 0.05 were considered statistically significant.

### 3.5. Drug Loading Procedure and Release Test

Firstly, SA was loaded onto aMMC by dissolving 400 mg of SA in 40 mL ethanol, to which 3.6 g of aMMC was added, thus aiming for a SA concentration of 10 wt.%. The mixture was placed on a shaker for 24 h at room temperature and then dried at 70 °C to let the solvent evaporate. Finally, the formulation was stored at 70 °C in order to avoid adsorption of moisture.

The release of SA from aMMC and the dissolution of pure SA was analyzed using a USP-2 dissolution bath (Sotax AT7 Smart, Sotax AG, Aesch, Switzerland) equipped with 1000 mL vessels (37 °C, 50 rpm) and paddles as stirring elements. Samples with a total content of 80 mg SA were placed in the vessels containing 1000 mL phosphate buffer (pH 6.8). Aliquots of 2 mL were withdrawn from each vessel at regular time intervals for 120 min and filtered through 0.8 µm surfactant-free cellulose acetate membrane filters (Minisart^®^ NML, VWR International, Spanga, Sweden) prior to analysis. The SA concentration was analyzed using a UV absorbance spectrophotometer at 296 nm. The aliquots were returned to the vessels after each time measurement. The measurements were made in triplicates on pure SA and on SA loaded in aMMC.

## 4. Conclusions

Salicylic acid was successfully loaded in aMMC to form a formulation containing 8 wt.% of the drug. This drug was subsequently released from aMMC in vitro in less than 15 min. Analyses of nitrogen sorption isotherms together with XRD and DSC results indicated that the SA was loaded into the pore structure of aMMC. Furthermore, SA was found to be present in its amorphous state and no reaction with the substrate was detected. The cytotoxicity test showed that aMMC did not exhibit toxicity toward hDF cells for all tested concentrations and exposure times. These results open up for further investigations of employing aMMC together with SA in topical formulations targeting different types of skin conditions. This work also demonstrates the possibility of formulating other types of acidic compounds with aMMC.

## Figures and Tables

**Table 1:** Specific surface area (S_BET_) and pore properties of the studied samples.

	S_BET_ (m^2^/g)	Pore Diameter (nm)	Pore Volume (cm^3^/g)	Particle Size (μm) ^a^
MMC	519	6.7	1.05	270
aMMC	509	5.5	0.70	19
aMMC-SA	441	5.5	0.57	-

MMC: mesoporous magnesium carbonate, aMMC: amine functionalized MMC, aMMC-SA: aMMC loaded with salicylic acid. ^a^ Average particle size from Figure S1 in Supplementary Materials.

## Supplementary Materials

The following are available online. Figure S1. Size distribution of MMC and aMMC. Evaluation of effect of pore volume. Table S1. Experimental and theoretical pore volume before and after loading of SA on aMMC.
