# Supplementary material for: Exploring the Use of Amine Modified Mesoporous Magnesium Carbonate for the Delivery of Salicylic Acid in Topical Formulations: In Vitro Cytotoxicity and Drug Release Studies
Source: Molecules. 2019 May 11;24(9):1820. doi: 10.3390/molecules24091820 (PMC6539276; doi:10.3390/molecules24091820)
Supplement: Supplementary file 1 [file molecules-24-01820-s001.pdf]

Supplementary Materials

Exploring the Use of Amine Modified Mesoporous Magnesium Carbonate for the Delivery of Salicylic Acid in Topical Formulations: In Vitro Cytotoxicity and Drug Release Studies

Maria Vall [1], Natalia Ferraz [1], Ocean Cheung [1], Maria Strømme [1] and Teresa Zardán Gómez de la Torre [1,*]

Division for Nanotechnology and Functional Materials, Department of Engineering Sciences, SE-751 21 Uppsala, Sweden; maria.vall@angstrom.uu.se (M.V.); natalia.ferraz@angstrom.uu.se (N.F.); ocean.cheung@angstrom.uu.se (O.C.); maria.stromme@angstrom.uu.se (M.S.)

* Correspondence: Teresa.Zardan@angstrom.uu.se; Tel.: +46-18-471-3381

Particle size characterization

The size distribution of powder samples containing either MMC or aMMC was evaluated using a Mastersizer 3000 equipped with a Hydro EV wet sample dispersion unit (Malvern Panalytical Nordic AB) at 25 °C. The sample was added to the dispersion unit, containing EtOH, until the desired obscuration range was reached and a stable scattering pattern was observed. Thereafter the measurement was started. All measurements were made in triplicates.

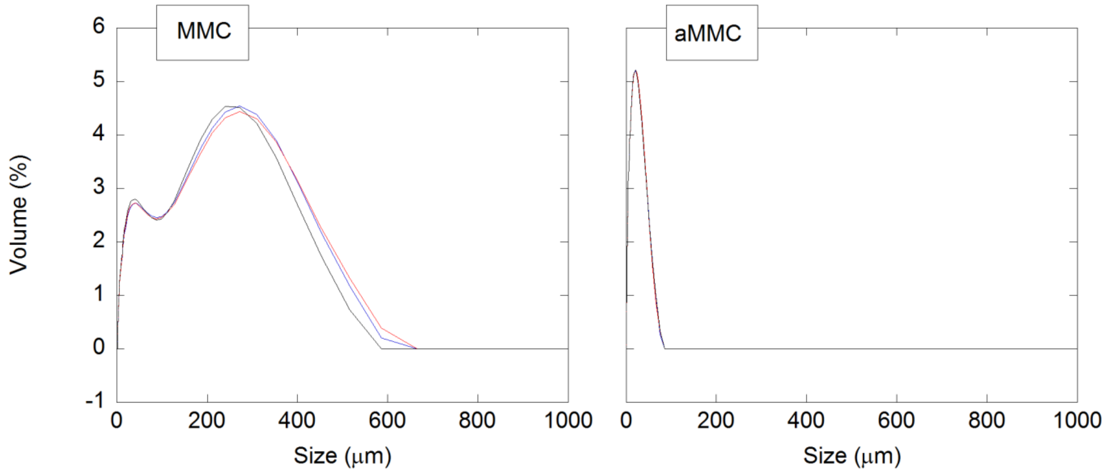

Figure S1. Size distribution of powder samples containing either MMC (left panel) or aMMC (right panel). All measurements were made in triplicates and are shown in the figure panels.

Evaluation of effect of pore volume

The theoretical effect that loading of SA inside or outside the pores of aMMC would have on the pore volume was calculated. When loaded on the surface of aMMC, the pores on aMMC would be blocked and prevent N_2 molecules from entering. As a result, the pore volume in such case would reduce significantly to a value close to zero. In the case where aMMC is in a mixture with 8 wt.% SA, the detected pore volume of the sample would decrease due to the increase in mass associated with the presence of SA. When SA is loaded inside the pores of aMMC, the volume of the loaded SA would effectively decrease the pore volume (per mass). The theoretical value was then calculated by deducting the volume of the loaded API, calculated from the density of SA (1.44 g/cm^3), from the pore volume before loading.

Table S1. Experimental and theoretical pore volume before and after loading of SA on aMMC.

Pore volume aMMC before loading (cm^3/g)	Amount loaded SA from TGA(wt.%)	Theoretical pore volume, when SA exists in a mixture with aMMC (cm^3/g)	Theoretical pore volume, SA loaded inside pores (cm^3/g)	Experimental pore volume, aMMC-SA (cm^3/g)
0.70	8	0.64	0.59	0.57
